# Thermosensitive TRPM2: The regulatory mechanisms of its temperature sensitivity and physiological functions

**DOI:** 10.1016/j.jphyss.2025.100008

**Published:** 2025-01-31

**Authors:** Makiko Kashio

**Affiliations:** Department of Cell Physiology, Faculty of Life Sciences, Kumamoto University, Japan

**Keywords:** TRPM2, Temperature

## Abstract

Transient receptor potential melastatin 2 (TRPM2) is a non-selective cation channel with high Ca^2+^ permeability. TRPM2 exhibits temperature sensitivity, detecting warm to noxious high temperatures. This temperature sensitivity is regulated by several endogenous factors, including reactive oxygen species, adenosine diphosphate ribose, Ca^2+^ ions, and TRPM2 phosphorylation by protein kinase C, which alter TRPM2 activity at body temperature. Consequently, at core body temperature, TRPM2 regulates the physiological functions of TRPM2-expressing cells and tissues, such as immunocytes, pancreatic β cells, and the brain. In contrast, TRPM2 in sensory neurons detects warm temperatures. The current review summarizes the regulatory mechanisms of TRPM2 and its roles in physiological processes, focusing on temperature-dependent phenomena.

## Background

Transient receptor potential (TRP) proteins constitute a superfamily of nonselective cation channels. Based on homology of the primary amino acid sequences, the TRP channel superfamily is divided into seven subfamilies: TRPV (vanilloid), TRPC (canonical), TRPM (melastatin), TRPML (mucolipin), TRPN (NompC), TRPP (polycystin), and TRPA (ankyrin). TRP channels are activated by various factors, such as chemical compounds and physical stimuli. TRP channels form tetramers, of which the subunits share a common structure of six transmembrane domains, with the ion-permeating pore between the fifth and sixth transmembrane domains forming a functional channel. Most TRP channels have Ca^2+^ permeability, and the activation causes membrane depolarization and cytosolic Ca^2+^ elevation. Transient receptor potential vanilloid 1 (TRPV1) is the founding member of the mammalian thermo-TRP channels, and this protein is activated by high temperatures (> 43 °C) that cause painful sensations [1]. Thereafter, TRPV1 was shown to function in sensory neurons to detect innocuous warm temperatures (36 °C-42 °C) in addition to noxious heat [2]. The discovery of TRPV1 prompted the exploration of other temperature-sensitive molecules; to date, 11 TRP channels have been identified as thermo-TRPs [3]. Each thermo-TRP is activated by a range of characteristic temperatures that represent activation thresholds. Thermo-TRPs cooperatively cover a wide range of temperatures, from noxious cold to noxious heat. On the other hand, it remains unclear whether temperature constitutes a physiologically relevant stimulus for all thermo-TRPs in vivo. Recent studies have clarified that the temperature thresholds for thermo-TRPs are regulated by various mechanisms, and thermo-TRP activity can be regulated at constant physiological temperatures.

Transient receptor potential melastatin 2 (TRPM2) is a thermo-TRP that detects warm to noxious high temperatures. Even though TRPM2 typically functions in the plasma membrane, several reports showed that TRPM2 also functions in the lysosomal membrane [4], [5]. While TRPM2 is expressed in sensory neurons [6], it is expressed mostly in cells and tissues, including the brain, pancreas, and immunocytes [7]. TRPM2 activity is thought to be regulated by physiological body temperature within the circadian fluctuation and hyperthermia caused by infection and inflammation; thus, TRPM2 participates in a wide range of physiological functions. Indeed, only a slight temperature increase from non-febrile to febrile is enough to augment cytosolic Ca^2+^ concentration in mouse macrophages in the presence of hydrogen peroxide (H_2_O_2_), a TRPM2 activator [8].

This review will summarize the current understanding of how TRPM2 function is regulated, and how the temperature-dependent activity of TRPM2 regulates physiological functions.

## Regulatory mechanisms of TRPM2 activity

TRPM2 is a non-selective cation channel with a broad expression pattern. TRPM2 activity is regulated by reactive oxygen species (ROS) [9], the intracellular endogenous agonist adenosine diphosphate ribose (ADPR) [10], and cytosolic Ca^2+^ ions [11]. Due to its ROS sensitivity, TRPM2 has been extensively studied for its involvement in cell death, including apoptosis and necrosis [12]. Most reports on TRPM2 indicate a deleterious role of TRPM2 in aggravating cell death in various pathological models [13].

TRPM2 activation by ROS occurs via indirect (mediated by cytosolic ADPR production) and direct mechanisms. One indirect pathway of ADPR production is mediated by nuclear poly (ADPR) polymerases (PARPs) and poly (ADPR) glycohydrolases (PARGs). PARP is activated by ROS to generate poly (ADPR) chains, which have DNA repair functions [14]. PARG hydrolyzes poly-ADPR to generate free ADPR. The significance of the PARP/PARG pathway in ROS-induced TRPM2 activation is supported by the finding that PARP inhibitors abrogate ROS-induced TRPM2 activation and cell death [15]. Another possible source of ROS-induced ADPR generation is mitochondria, where ROS stimulate nicotinamide adenine dinucleotide (NAD^+^) release and ADPR generation [16]. In contrast, direct activation of TRPM2 is also involved in ROS-mediated activation of TRPM2 [8]. Heat-evoked activation of TRPM2 is markedly enhanced by ROS, and this was detected under inside-out single-channel recording conditions, in which intracellular organelles are absent.

Cytosolic ADPR is well established as an endogenous TRPM2 activator [10]. In addition, ADPR analogs (2’/3’-o-acetyl ADPR and 2-deoxy ADPR) also activate TRPM2 [17], [18]. TRPM2 activation by 2-deoxy ADPR is reportedly more effective and requires a lower concentration of cytosolic Ca^2+^ compared with activation by ADPR [18]. TRPM2 was once considered to be activated by pyridine dinucleotides such as NAD^+^, nicotinic acid adenine dinucleotide (NAAD^+^) and NAAD-2’-phosphate, and cyclic ADPR (cADPR). However, TRPM2 activation by these compounds is probably due to ADPR contamination in commercial reagents, and therefore pyridine dinucleotides and cADPR are likely not TRPM2 agonists [19], [20]. Moreover, cytosolic Ca^2+^ plays an indispensable role in TRPM2 activation, and TRPM2 is not activated in the complete absence of extracellular and cytosolic Ca^2+^ [11]. TRPM2 activation in the presence of extracellular Ca^2+^ allows Ca^2+^ influx into the cytosol, and an elevated cytosolic Ca^2+^ concentration augments TRPM2 channel activity, forming a positive feedback loop. Moreover, the Ca^2+^ sensitivity of TRPM2 may be regulated by phosphatidylinositol 4,5-bisphosphate in the inner membrane leaflet [21].

## Temperature-dependent activation of TRPM2

Temperature is another factor regulating TRPM2 activity. Many thermo-TRPs show apparent temperature thresholds for their activation. TRPM2 was originally reported to have a temperature threshold of approximately 37 °C [22]. However, the temperature threshold of TRPM2 is now known to vary under different scenarios. The temperature threshold for TRPM2 activation is ∼47 °C when heterologously expressed in HEK293T cells [8], and this threshold can be modulated to enable TRPM2 regulation under physiological temperatures. H_2_O_2_, a type of ROS, decreases the threshold of TRPM2 to physiological temperatures (< 37 °C), enabling channel activation at these temperatures. The effect of H_2_O_2_ on the temperature sensitivity of TRPM2 is observed even in excised membrane patches lacking intracellular organelles, such as nuclei and mitochondria, which are involved in ROS-induced ADPR generation [15], [16]. In addition, an elevation in temperature from 14 °C to 40 °C increases TRPM2 sensitivity to ADPR and cytosolic Ca^2+^. The resulting increase in TRPM2-mediated Ca^2+^ influx can thereby act synergistically at a Ca^2+^ binding site on the cytosolic side just underneath the pore of the channel to further decrease the temperature threshold and further increase TRPM2 activity, in a positive feedback loop [23].

**Fig. 1:**
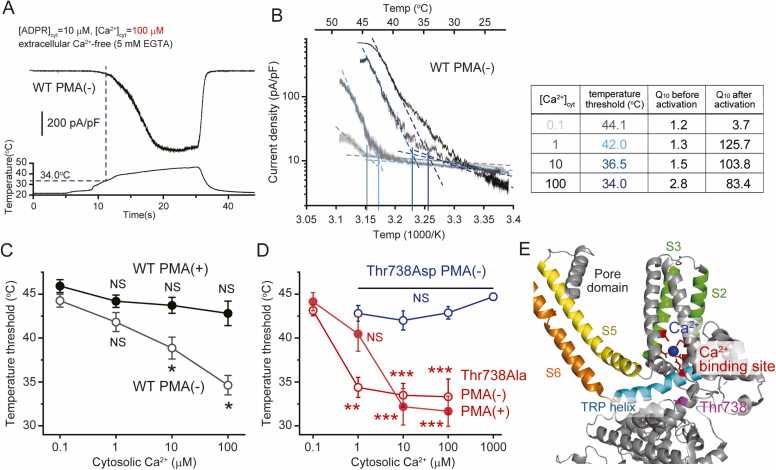
Temperature threshold of TRPM2 regulated by cytosolic Ca^2+^ and phosphorylation via PKC. (A) Heat-evoked TRPM2 currents in the presence of cytosolic ADPR (10 µM) and Ca^2+^ (100 µM) in the absence of extracellular Ca^2+^ (5 mM EGTA). (B) Left: Arrhenius plots of heat-evoked TRPM2 currents in the presence of cytosolic Ca^2+^ (0.1, 1, 10, and 100 µM). Right: calculated temperature thresholds and Q_10_ values before and after heat-evoked TRPM2 current activation. (C) Cytosolic Ca^2+^-dependent reduction in the TRPM2 threshold abrogated by pretreatment with the PKC activator PMA. Heat-evoked TRPM2 currents were recorded 2–3 h after pretreatment with PMA (100 nM, 20 min). ^NS^p ≥ 0.05, *p < 0.05 vs. 0.1 µM Ca^2+^ (Bonferroni test following two-way ANOVA). (D) Abrogation of the PMA-induced threshold elevation by a phospho-deficient mutation (Thr738Ala), and reproduction of the effect of PMA on WT TRPM2 by a phospho-mimicking mutation (Thr738Asp). ^NS^p ≥ 0.05, * *p < 0.01, * **p < 0.001 vs. 0.1 µM Ca^2+^ (Bonferroni test following two-way ANOVA). (E) Predicted location of Thr738 in the tertiary structure of mouse TRPM2 (adapted human TRPM2, PDB ID; 6pus). Thr738 (T738, magenta); Ca^2+^ (blue); Ca^2+^ binding pocket (red) formed by the TRP helix (cyan), S2 and S3 (green); S5 (yellow) and S6 (orange) of a single subunit are highlighted.

In addition, cytosolic Ca^2+^ lowers the temperature threshold of TRPM2 activation in a concentration-dependent manner (Fig. 1A–C), suggesting that TRPM2 is regulated by the signals that mobilize cytosolic Ca^2+^. Temperature coefficient (Q_10_) values showed ∼100-fold increase after heat-evoked activation of TRPM2 under fixed cytosolic Ca^2+^ concentrations of 1, 10, and 100 µM, during the absence of extracellular Ca^2+^ (Fig. 1B) [24]. These results indicate intrinsic temperature sensitivity of TRPM2 regardless of cytosolic Ca^2+^ elevation through activation of the TRPM2 pore. Interestingly, the abovementioned effect of cytosolic Ca^2+^ on the TRPM2 threshold was counteracted by TRPM2 phosphorylation via protein kinase C (PKC), as phosphorylation elevates the TRPM2 threshold (Fig. 1C). Considering these results, threshold elevation by PKC may occur following cytosolic Ca^2+^ elevation as negative feedback regulation of TRPM2. PKC-mediated regulation of the TRPM2 threshold was completely abolished by the phosphorylation-deficient mutation Thr738Ala and was recapitulated by the phosphorylation-mimicking mutation Thr738Asp (Fig. 1D). The threonine residue (Thr738) responsible for threshold regulation by PKC was predicted to be near the cytosolic Ca^2+^-binding site of TRPM2 (Fig. 1E). These results suggest that phosphorylation of Thr738 introduces bulky negative charges and could affect the Ca^2+^ affinity for Ca^2+^-binding sites, to counteract the effect of cytosolic Ca^2+^ on the temperature sensitivity of TRPM2.

## Physiological functions of TRPM2

### Immunity

TRPM2 is widely expressed in leukocytes, including lymphocytes, neutrophils, monocytes/macrophages, dendritic cells, and microglia [4], [8], [25], [26], [27], [28]. Therefore, many studies have reported TRPM2 involvement in immune reactivity and inflammation. At infection sites, pathogenic agents are engulfed by phagocytes, such as neutrophils and macrophages, and then sterilized via ROS production by NADPH oxidase (Nox) in phagosomes. Many reports have shown that TRPM2 activation by ROS aggravates inflammation by elevating inflammatory cytokine release. TRPM2 activation in monocytes increases the release of neutrophil-attracting chemokine (C-X-C motif) ligand 2 (CXCL2) [27]. Indeed, TRPM2KO mice showed attenuated CXCL2 levels and neutrophil infiltration in inflamed colon tissues in an experimental model of dextran sulfate sodium-induced ulcerative colitis. These results suggest that TRPM2 activation in monocytes promotes CXCL2 release and neutrophil migration toward inflamed tissues to aggravate inflammation. Moreover, TRPM2 elevates the release of cytokines from macrophages: granulocyte colony stimulating factor, interleukin (IL)−1α, and CXCL2 [8].

In contrast, beneficial functions of TRPM2 in enhancing host defense have been reported in several infection/inflammation models. An in vivo study of *Listeria monocytogenes* (*Lm*) infection showed that TRPM2 deficiency attenuates serum interferon gamma (IFNγ)/IL-12p40 levels and iNos^+^ monocyte numbers in tissues and elevates the tissue bacterial burden [29]. Moreover, *Lm* infection increased the mortality rate of TRPM2KO mice. The enhanced susceptibility of TRPM2KO mice was largely recovered by application of recombinant IFNγ, suggesting that TRPM2 plays a role upstream of the IFNγ receptor. A recent report using the same in vivo *Lm* infection model showed higher susceptibility of TRPM2KO mice to infection but no difference in the serum IFNγ level between wild-type (WT) and TRPM2KO mice [30]. Instead, *Lm*-infected TRPM2KO mice had higher serum levels of cytokines including tumor necrosis factor alpha (TNFα), IL-6, IL-10, and C-C motif chemokine ligand 2 (CCL2), with increased numbers of neutrophils and monocytes in infected tissues. In the same report, in an in vitro *Lm* infection study, TRPM2KO macrophages and neutrophils released higher levels of proinflammatory IL-1β and IL-1α, but TNFα release was comparable between WT and TRPM2KO cells. TRPM2KO neutrophils exhibited higher ROS production and bactericidal activity against *Lm* compared with WT neutrophils. These data suggest that TRPM2 deficiency causes hyperinflammatory activity during *Lm* infection. Hyperinflammatory reactions in TRPM2KO mice were also reported in a *Helicobacter pylori* infection model probably due to TRPM2-dependent suppression of ROS production [31].

TRPM2 has a reported role in lipopolysaccharide (LPS)-induced inflammation as well. TRPM2 downregulation by shRNA suppressed LPS-induced release of IL-6, IL-8, IL-10, and TNFα from THP-1 human monocytes [32]. In contrast, an in vivo LPS-induced lung inflammation model showed higher mortality with pronounced lung edema in TRPM2KO mice than in WT mice. In addition, lung CXCL2, IL-6, TNFα, and myeloperoxidase activities were elevated in TRPM2KO mice [33]. LPS-induced ROS production was markedly enhanced in TRPM2KO neutrophils, consistent with increased LPS-induced TNFα release. As activity of the electrogenic Nox enzyme is known to be voltage-sensitive [34], membrane depolarization through TRPM2 is considered to suppress Nox activity, and the absence of this regulatory mechanism in TRPM2KO mice could enhance ROS production and aggravate inflammation.

Many inflammatory cascades, such as IL-1β processing and release, depend on inflammasome activation. Nod-like receptor family pyrin domain containing-3 (NLRP3) inflammasomes are activated by several conditions of cellular stress, including microbial products, reduced intracellular K^+^ concentration, and increased intracellular Ca^2+^ concentration ([Ca^2+^]_i_) [35], [36]. TRPM2 is reportedly involved in inflammasome activation in macrophages and monocytes [37], [38].

TRPM2 activity is reportedly involved in phagocytosis, an important process in innate immunity responsible for removing pathogens. Phagocytes such as macrophages and neutrophils engulf deleterious agents and digest them in phagosomes by producing ROS via Nox activity. Phagocytosis is enhanced by febrile temperatures [39]. The toll-like receptor 2 agonist zymosan stimulates phagocytosis by macrophages and ROS production. This phagocytic activity is amplified at febrile temperatures in WT macrophages but not in TRPM2KO cells [8]. Consistently, slight temperature elevation from non-febrile to febrile temperature augments cytosolic Ca^2+^ signals in mouse peritoneal macrophages treated with a low concentration of H_2_O_2_, suggesting that ROS and febrile temperatures synergize to activate TRPM2 to increase phagocytosis. Moreover, TRPM2 is highly localized in phagosomal membranes, and functional coupling between TRPM2 and Nox through phagosomal acidification may be involved in the regulation of phagocytic activity and pathogen clearance [40].

Immunocytes have a high migratory ability, known as chemotaxis, which is elevated in a temperature-dependent manner [39]. TRPM2 has a role in immunocyte chemotaxis. H_2_O_2_ has been reported to drive neutrophil chemotaxis [41], and WT neutrophils showed chemoattraction to H_2_O_2_ at a concentration range of 1 nM to 10 µM [42]. TRPM2KO in neutrophils almost completely abolished this chemoattraction, suggesting an indispensable role of TRPM2 in H_2_O_2_-induced chemoattraction. Conversely, a higher concentration of H_2_O_2_ (100 µM) inhibited chemoattraction. Interestingly, chemoattraction of WT neutrophils was elevated in a temperature-dependent manner (33–41 °C) when chemoattraction was stimulated not only by a low concentration of H_2_O_2_ (1–10 nM) but also by chemokines (CXCL2), complement (C5a), and LPS. Conflicting inhibitory and acceleratory effects of TRPM2 in neutrophil chemotaxis toward the bacterial formyl peptide fMet-Leu-Phe, which stimulates ROS generation, have been reported [42], [43]. Stimulation versus inhibition of chemoattraction by H_2_O_2_ seems to depend on its concentration, suggesting that TRPM2 activity exerts biphasic regulation of chemotaxis. Dendritic cell chemotaxis by chemokines CXCL12 and CCL19 is reportedly augmented by TRPM2 activity [4]. Microglia are glial cells with immune reactivity in the central nervous system and spinal cord. The migratory activity of brain microglia is enhanced by TRPM2 in a temperature-dependent manner [44].

### Insulin secretion

**Fig. 2:**
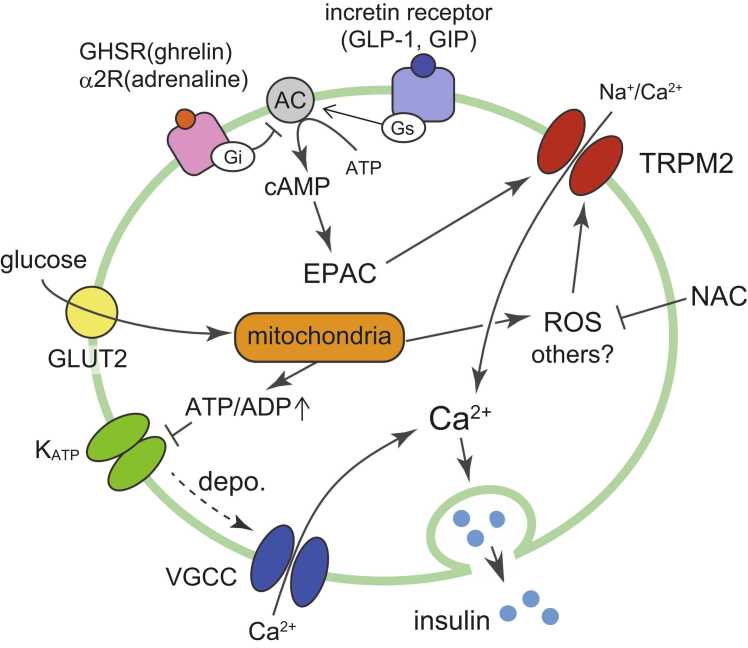
Functional involvement of TRPM2 in insulin secretion from pancreatic β-cells. Blood glucose is transported into pancreatic β cells via glucose transporter 2 (GLUT2) and is metabolized, thereby increasing the ATP/ADP ratio. The increased ATP/ADP causes the K_ATP_ channel to close, leading to depolarization (depo.) and activation of voltage-gated Ca^2+^ channels; the subsequent elevation in intracellular [Ca^2+^] leads to insulin secretion. Reactive oxygen species (ROS) generated by glucose metabolism increase TRPM2 activity at body temperature. The antioxidant N-acetyl cysteine (NAC) suppresses ROS activity and glucose-stimulated insulin secretion. Activation of the Gs-coupled incretin receptor leads to exchange protein directly activated by cAMP (EPAC) activation via cAMP generation by adenylate cyclase (AC). EPAC elevates insulin secretion through TRPM2 activity. The Gi-coupled growth hormone secretagogue receptor (GHSR) and adrenaline α2 receptor pathways counteract the EPAC pathway to suppress insulin secretion.

Insulin is the hormone responsible for decreasing the blood glucose level. The rapid increase in blood glucose after meals evokes insulin secretion from pancreatic β cells. The released insulin is carried via the bloodstream to the liver, skeletal muscles, and adipose tissues to enhance glucose uptake into these organelles, leading to a decrease in blood glucose. Glucose-stimulated insulin secretion (GSIS) is mediated by a pathway involving ATP-sensitive K^+^ (K_ATP_) channel closure via elevation of the intracellular ATP/ADP ratio following glucose metabolism (Fig. 2). Decreased K_ATP_ activity leads to membrane depolarization and activation of a voltage-gated Ca^2+^ channel. Depolarization and [Ca^2+^]_i_-elevation via non-selective cation channels is also an amplification mechanism of GSIS. TRPM2 is expressed in pancreatic β cells and contributes to non-selective cation channel activity to enhance GSIS.

TRPM2KO mice show attenuated insulin secretion and higher blood glucose levels in an in vivo glucose tolerance test compared with WT mice [45]. Consistently, GSIS from pancreatic islets was attenuated by TRPM2 deficiency. ROS are produced in pancreatic β cells downstream of physiological mediators, including blood glucose [46], [47]. Therefore, ROS might increase TRPM2 activity at body temperature to enhance insulin secretion. Indeed, the antioxidant N-acetyl cysteine (NAC) attenuates GSIS in WT islets but not in TRPM2KO islets [48]. Temperature elevation from 33 °C to 40 °C increased GSIS from WT islets. The inhibitory effect of NAC on GSIS was amplified at higher temperatures in WT islets; however, this temperature-dependent amplification was completely abolished in TRPM2KO islets. These data suggest that TRPM2 activation elevates GSIS in a temperature- and ROS-dependent manner. Moreover, TRPM2 contributes to hormone-regulated insulin secretion (Fig. 2). Glucagon-like peptide-1 and gastric inhibitory polypeptide are incretin hormones with hypoglycemic activity that are released from the intestine following meals. Incretin hormones synergistically enhance GSIS from pancreatic islets with blood glucose elevation [49]. Incretin activates Gs-coupled incretin receptors and activation of adenylate cyclase to generate cAMP. The elevated cAMP activates the exchange protein directly activated by cAMP (EPAC), which increases GSIS in a TRPM2-dependent manner [45], [50]. The EPAC pathway is inhibited by ghrelin, a hormone released from the stomach during fasting. Activation of a Gi-coupled ghrelin receptor, growth hormone secretagogue receptor, counteracts the Gs-coupled EPAC receptor pathway, thereby decreasing GSIS [51]. Sympathetic activity by adrenaline α2 receptor activation in pancreatic islets has the same effect as ghrelin in decreasing GSIS [52].

In contrast, inflammation induced by TRPM2 causes insulin resistance in peripheral tissues [53]. TRPM2KO mice show lower susceptibility to insulin resistance and obesity induced by a high-fat diet, as well as lower cytokine levels and macrophage infiltration in adipose tissue, compared with WT mice [53]. Energy expenditure and glucose uptake in the muscles and heart are higher in TRPM2KO mice than in WT mice. These data suggest that, in addition to regulating insulin secretion, TRPM2 is involved in regulating insulin sensitivity in glucose-consuming tissues.

### Thermosensation

TRPM2 is expressed in a small population of heat-sensitive peripheral sensory neurons and autonomic neurons (novel heat-sensitive neurons) that have no sensitivity to either TRPV1 or TRPM3 activators when assessed in sensory neuron cultures [6]. The temperature sensitivity of these novel heat-sensitive neurons showed characteristic features of TRPM2 sensitization by H_2_O_2_ [8], and the response was decreased in TRPM2KO neurons. Therefore, TRPM2 is suggested to act as a sensor of warm temperature in peripheral sensory neurons. On the other hand, another study, which used mice lacking all TRPV1, TRPM3 and TRPA1 (triple-KO), found that triple-KO sensory neurons failed to show TRPM2-like heat sensitivity [54]. The discrepancy between reports might reflect the effect of the cell culture process on temperature sensitivity of sensory neurons. In the in vivo thermal preference test, TRPM2KO mice could not sense a difference between 33 °C and 38 °C, while WT mice showed a preference for 33 °C [6]. A recent study recapitulated the thermal preference behavioral deficit of TRPM2KO mice, which preferred significantly warmer temperatures compared to littermate controls [55]. However, this study did not observe any substantial TRPM2 phenotype in cultured sensory neurons, leaving the question unresolved as to how TRPM2 mediates thermal preference.

Similar to TRPM2, its orthologue TRPM8 plays a role in the sensation of innocuous warm temperatures [56]. TRPM8 is activated by cool temperatures (< 27 °C) and menthol [57], [58], both of which cause cool sensations in humans. However, the temperature sensitivity of TRPM8 seems to depend on the cellular environment. A slight temperature decrease from physiological temperature (35 °C) caused a substantial Ca^2+^ increase and spontaneous firing in trigeminal ganglion neurons [59]. Conversely, TRPM8 activity at physiological temperature is suppressed by temperature elevation and is involved in detection of innocuous warmth. In an *ex vivo* skin-nerve preparation study, TRPM8KO tissues lacked “warmth-inhibited” neural populations [56]. Moreover, in an in vivo study, WT mice successfully detected slight (> 1 °C) temperature elevations of palm surface from 22 or 33 °C, while TRPM8KO mice failed to respond to the temperature elevation. As the warmth sensitivity detected in the same analysis was also affected in TRPM2KO mice, TRPM2 and TRPM8 are thought to be involved in warmth perception. In the thermal ring gradient test, TRPM8KO mice showed a significantly affected temperature preference, with prolonged staying time in noxious cold temperatures (< 20 °C) and decreased time in innoxious warm temperature (∼35 °C) compared with WT mice [60]. In contrast, TRPM2KO mice showed no obvious difference in thermal preference. Therefore, further studies are necessary to determine the significance of TRPM2 in warmth sensation. Warm temperature sensation by TRPM2 and TRPM8 was also involved in temperature-dependent masking behavior, a direct behavioral response to environmental changes [61].

### Body temperature regulation

Under fluctuating environmental temperatures, endothermic species such as mammals and birds need to maintain their body temperature in the optimal range for survival. To achieve this thermoregulation, the hypothalamic preoptic area (POA) receives peripheral temperature information from the skin and abdomen via sensory neurons. Moreover, the POA harbors warmth-sensitive neurons with intrinsic temperature sensitivity to monitor the changes in local brain temperature [62]. Consequently, the POA integrates peripheral and core body temperatures and functions as a thermoregulating center to drive autonomic and behavioral responses to control body temperature [63]. Local warming of the POA induces cutaneous vasodilation to increase heat dissipation and inhibits shivering behavior [64]. In contrast, POA cooling causes vasoconstriction of the tail vein, and elevations in oxygen consumption and core body temperature [65]. These results suggest that local POA warming and cooling drives heat and cold adaptation to regulate thermogenesis and heat dissipation, further supporting the significance of the thermosensitivity of hypothalamic neurons in body temperature regulation.

POA neurons in mice express TRPM2 and are involved in thermoregulation, particularly in response to fever [66]. Heat (45 °C)-evoked responses in POA neurons are diminished by TRPM2 inhibitors. Moreover, such responses show characteristic features of TRPM2, such as sensitization by H_2_O_2_ treatment. POA neurons prepared from TRPM2KO mice lacked this response, confirming that the heat-evoked response is due to TRPM2 activation. Even though heterologously expressed TRPM2 shows a high temperature threshold [8], POA slice preparations in which synaptic connections were conserved showed a definite response to mild warm temperature (38 °C) [66]. Selective activation of TRPM2-expressing POA neurons by a designer receptors exclusively activated by designer drugs (DREADD) system caused hypothermia accompanied by decreased locomotor activity and cutaneous vasodilation of the tail vein. On the other hand, DREADD-mediated inhibition of TRPM2-expressing POA neurons caused hyperthermia, suggesting that activation and inhibition of TRPM2-expressing POA neurons drive heat dissipation and thermogenesis, respectively. Injection of prostaglandin E2 into the POA induced a larger febrile response in TRPM2KO mice compared with WT mice, suggesting that TRPM2 activity in POA neurons limits fever development. However, multiple pathways other than TRPM2 in the POA might ensure normal body temperature regulation, particularly since TRPM2KO mice exhibit a normal core body temperature with a diurnal rhythm at ambient temperature [66]. In addition, TRPM2 is expressed in sensory and autonomic neurons, and TRPM2KO mice prefer substantially warmer temperatures [6]. Considering that temperature selection capability is important for body temperature regulation, TRPM2 expression in sensory and autonomic neurons could affect body temperature in environments with unstable ambient temperatures. Moreover, TRPM2KO mice showed a weaker hypothermic response evoked by local hypothalamic heating compared with WT mice [67]. It is suggested that TRPM2 is a component of a temperature-dependent disinhibitory circuit, but that it does not function independently as a warmth sensor in warmth-sensitive neurons. Moreover, ultrasound-evoked TRPM2 activation in POA neurons was reported to drive hypothermia, which was attenuated in TRPM2KO mice [68], further suggesting TRPM2 roles in body temperature regulation.

A recent study showed that TRPM2 expression in vagal sensory neurons is regulated by the synaptic scaffold protein SHANK3, and SHANK3 deficiency affected the body temperature response in an LPS-induced systemic inflammation model [69]. SHANK3KO mice exhibited more severe hypothermia, a higher serum IL-6 level, and a higher mortality rate after LPS injection compared with WT mice. SHANK3KO nodose ganglion neurons showed lower TRPM2 expression but unaffected expression of another heat sensor, TRPV1. TRPM2 downregulation in nodose ganglion neurons recapitulated the LPS-induced hypothermia and elevated serum IL-6 level observed in SHANK3KO mice. However, further studies are needed to confirm the role of TRPM2 as a temperature sensor in vagal sensory neurons and its involvement in body temperature regulation.

## Structural basis of TRPM2 determining temperature sensitivity: a point of dispute

TRPM2 is conserved among a wide range of species, from protists to vertebrates. Several cryo-EM analyses have revealed the structures of human, zebrafish, sea anemone, and choanoflagellate TRPM2 in different conformational states [70], [71], [72], [73], [74], [75].

The cryo-EM structure of ADPR-bound human TRPM2 revealed two different ADPR-binding sites in the N-terminal MHR1/2 and C-terminal NUDT9H domains of each subunit, for a total of eight binding sites within a single TRPM2 channel molecule [74]. The C-terminal NUDT9H domain is homologous to that of the NUDT9 protein, a mitochondrial ADPR hydrolase that cleaves ADPR into adenosine monophosphate and ribose-5-phosphate. Therefore, TRPM2 has been considered a channel enzyme (chanzyme) with combined functions of ion permeation and enzyme activity. Indeed, the NUDT9H domains of *Nematostella vectensis* TRPM2 and *Salpingoeca rosetta* TRPM2 were shown to be enzymatically active [75], [76]. Removal of the C-terminal NUDT9H domain from *N. vectensis* TRPM2 failed to affect ADPR-evoked current activation [77], suggesting that channel activation is independent of this domain. In contrast, TRPM2 of more advanced species seems to have lost its NUDT9H enzymatic activity during molecular evolution [76]. As mentioned above, the cryo-EM structure of human TRPM2 revealed N-terminal and C-terminal ADPR binding sites [74]. On the other hand, the cryo-EM structure of zebrafish TRPM2 clearly showed ADPR binding to the N-terminal MHR1/2, but not NUDT9H, domain [71]. Therefore, ADPR may have higher affinity for the NUDT9H domain of human TRPM2 than of zebrafish TRPM2. The compound 8-Br-cADPR inhibits cADPR-evoked TRPM2 currents but not ADPR activity [78], and 8-Br-cADPR binds only to the N-terminal MHR1/2 domain of human TRPM2 [74]. These data suggest pivotal roles of the N-terminal MHR1/2 domain and assisting roles of the C-terminal NUDT9H domain in TRPM2 activation, even though NUDT9H is not necessary for activation of invertebrate TRPM2.

Ca^2+^ ions, another TRPM2-activating factor, interact with the Ca^2+^ binding domain located just beneath the transmembrane region of TRPM2. The Ca^2+^ binding domain of TRPM2 consists of the Glu843/Gln846/Asn869/Asp872 residues within transmembrane segments S2 and S3 and the Glu1073 residue within the TRP domain in human TRPM2. The Ca^2+^ binding domain is conserved among human, zebrafish, sea anemone, and choanoflagellate TRPM2 proteins [70], [71], [72], [75]. ADPR/Ca^2+^ binding to TRPM2 causes large structural changes in the intracellular region, which seem to affect the transmembrane region [74]. Moreover, the TRP domain consisting of the Ca^2+^ binding domain moves closer to the Ca^2+^ binding pocket upon channel activation [70]. The Thr738 residue responsible for phosphorylation-mediated TRPM2 threshold regulation might be located near the Ca^2+^ binding domain (Fig. 1E) [24]. Phosphorylation of this residue seems to affect its Ca^2+^ affinity to counteract the effect of cytosolic Ca^2+^ on the temperature threshold for TRPM2 activation. However, the results of cryo-EM studies were obtained at low temperature and thus do not reflect temperature-dependent activation of TRPM2. A rapid freezing strategy was reported to reveal the cryo-EM structure of the heat-activated state of thermo-sensitive TRPV3 and TRPM4 [79], [80]. Further studies are needed to clarify the heat-activated state of TRPM2 to determine how temperature changes cause TRPM2 activation.

## Funding

This work was supported by Grant-in-Aid for Scientific Research to MK from the 10.13039/501100001700Ministry of Education, Culture, Sports, Science and Technology in Japan (#20K06748), and Takeda Science Foundation, Medical Research Continuous Grants.

## CRediT authorship contribution statement

**Makiko Kashio:** Conceptualization, Writing – original draft, Writing – review & editing.
